# Ablation of Dihydroceramide Desaturase Confers Resistance to Etoposide-Induced Apoptosis In Vitro

**DOI:** 10.1371/journal.pone.0044042

**Published:** 2012-09-11

**Authors:** Monowarul M. Siddique, Benjamin T. Bikman, Liping Wang, Li Ying, Erin Reinhardt, Guanghou Shui, Markus R. Wenk, Scott A. Summers

**Affiliations:** 1 Program in Cardiovascular and Metabolic Diseases, Duke-National University of Singapore Graduate Medical School, Singapore, Singapore; 2 Department of Physiology and Developmental Biology, Brigham Young University, Provo, Utah, United States of America; 3 Sarah W. Stedman Nutrition and Metabolism Center, Duke University Medical Center, Durham, North Carolina, United States of America; 4 Department of Biochemistry, National University of Singapore, Singapore, Singapore; Universidade Federal do Rio de Janeiro, Brazil

## Abstract

Sphingolipid biosynthesis is potently upregulated by factors associated with cellular stress, including numerous chemotherapeutics, inflammatory cytokines, and glucocorticoids. Dihydroceramide desaturase 1 (Des1), the third enzyme in the highly conserved pathway driving sphingolipid biosynthesis, introduces the 4,5-trans-double bond that typifies most higher-order sphingolipids. Surprisingly, recent studies have shown that certain chemotherapeutics and other drugs inhibit Des1, giving rise to a number of sphingolipids that lack the characteristic double bond. In order to assess the effect of an altered sphingolipid profile (via Des1 inhibition) on cell function, we generated isogenic mouse embryonic fibroblasts lacking both *Des1* alleles. Lipidomic profiling revealed that these cells contained higher levels of dihydroceramide than wild-type fibroblasts and that complex sphingolipids were comprised predominantly of the saturated backbone (e.g. sphinganine vs. sphingosine, dihydrosphingomyelin vs. sphingomyelin, etc.). *Des1* ablation activated pro-survival and anabolic signaling intermediates (e.g. Akt/PKB, mTOR, MAPK, etc.) and provided protection from apoptosis caused by etoposide, a chemotherapeutic that induces sphingolipid synthesis by upregulating several sphingolipid biosynthesizing enzymes. These data reveal that the double bond present in most sphingolipids has a profound impact on cell survival pathways, and that the manipulation of Des1 could have important effects on apoptosis.

## Introduction

Sphingolipids are comprised of a sphingosine backbone coupled to a fatty acyl-chain. *De novo* sphingolipid biosynthesis involves a highly conserved pathway initiated by the condensation of palmitate and serine. Four sequential reactions, which occur in the endoplasmic reticulum and possibly mitochondria [1], lead to the formation of ceramide, the precursor for complex sphingolipids such as sphingomyelin and gangliosides [2]. A number of chemotherapeutics have been shown to induce sphingolipid synthesis, which is the likely mechanism responsible for suppression of tumor growth [3]. In recent years, various sphingolipids (e.g. ceramides, dihydroceramides, sphingosine, sphinganine, ceramide 1-phoshate, sphingosine 1-phosphate, etc.) have been implicated in the regulation of cell growth, death, and metabolism [4], [5], [6], [7], [8].

The third reaction in the ceramide biosynthesis pathway is catalyzed by dihydroceramide desaturase, an enzyme that introduces a 4,5-trans-double bond into the sphinganine backbone of dihydroceramide. Surprisingly, lipidomic analysis has recently revealed that a number of factors including various chemotherapeutics [9], [10], reactive oxygen species [11], and resveratrol [12] inhibit dihydroceramide desaturation, promoting accumulation of the dihydro form of high-order sphingolipids. These observations have prompted studies on this enzyme and this unique class of lipids [12], [13].

In the majority of tissues, the double bond is introduced by dihydroceramide desaturase 1 (Des1). The second isoform, Des2, is highly enriched in epithelial cells (gut, kidneys, skin), but is largely absent from other tissues [14]. Des1 displays specificity for synthesis of ceramide [15], while Des2 produces both ceramides and phytoceramides [16], [17]. We have previously described a knockout mouse (*Des1^−/−^*) devoid of Des1 [18] which demonstrated a plethora of growth abnormalities and tremors, ultimately proving lethal at about 8-weeks of age. Thus, these findings highlight the importance of the double bond in the sphinganine backbone in normal cellular function. However, the molecular basis for its necessity has been enigmatic.

Previous studies that assessed the role of dihydrosphingolipids in cellular function utilized one of three approaches. First, dihydroceramides were added exogenously to cultured cells [9], [14], [19], [20]. Second, small interfering RNAs were transfected into cells to achieve a partial knockdown of the enzyme [13], [21]. Third, the effects of the aforementioned Des1 inhibitors were assessed [12], [13]. While each of these approaches has made significant contributions to our understanding of sphingolipid biology, they have inherent limitations. Incomplete or transient inhibition of Des1 and the extreme difficulties with synthetic dihydroceramide solubility make it difficult to study. Moreover, many of the pharmacological inhibitors (e.g. fenretinide, resveratrol) are known to have multiple effectors. To gain a better understanding of the role of Des1 and dihydrosphingolipids in cellular stress responses, we studied cells completely devoid of Des1. The data reveal that Des1 ablation has a potent and dramatic effect on cell survival pathways.

## Materials and Methods

### MEF culture

Des1 +/+ or −/− MEFs were obtained from our previous studies done in University of Utah and Duke University, USA [18]. Briefly, mouse embryos were isolated from pregnant Des1 +/+ (C57BL/6 mice) or Des1 −/− mice (C57BL/6 mice lacking full-length dihydroceramide desaturase1), and the cells were immortalized by sequentially passing them in Dulbecco's modified Eagle's medium (DMEM) supplemented with 10% fetal calf serum (Hyclone, Thermo Scientific, USA) in a water jacket incubator in the presence of 7.5% CO2 at 37°C. The studies were conducted in accordance with the principles and procedures outlined in the National Institutes of Health Guide for the Care and Use of Laboratory Animals and were approved by the IACUCs at the University of Utah, and Duke University.

### MTS Assay to measure cell viability

The CellTiter 96® AQueous One Solution Cell Proliferation Assay Kit (Promega, USA) was used to measure the percentage of viable cells after etoposide or ceramide treatment according to the manufacturer's protocol. 24 hours before treatment, 5000 cells/well were placed in 96-well plates containing 100 µl of culture medium. Cells were treated with etoposide or ceramide as indicated in the Figure legends and 20 µl of CellTiter 96® AQueous solution was added to each well. Following a 4-hour incubation at 37°C in a humidified (7.5% CO_2_) incubator, absorbance at 490 nm was recorded using microplate reader (Infinite M200 PRO, TECAN, Switzerland).

### Analysis of cell death

#### Caspase activation

The Caspase-Glo® 3/7 Assay Kit (Promega, USA) was used to quantify activation of apoptotic caspases (caspase 3 & 7) according to the manufacturer's protocol. 24 hours before treatment, 5000 cells were plated per well in a 96-well plate containing 100 µl of culture medium. Cells were treated with etoposide or ceramide as indicated in the figure legend, and 100 µl of Caspase-Glo ® 3/7 assay solution was added. Following 4-hour incubation at 37°C in a humidified (7.5% CO_2_) incubator, fluorescence was recorded using microplate reader (Infinite M200 PRO, TECAN, Switzerland).

#### Propidium iodide Staining

Cell death was measured by staining cells with propidium iodide (PI) and detected with a fluorescence cell sorter (Cytomics FC500, Beckman Coulter, USA). Cells were trypsinized and were collected in 5 ml polystyrene round-bottom tubes and then centrifuged at 300 g. The cell pellets were washed twice with cold PBS. The pellet was resuspended in 500 µl of PBS and 25 µg of propidium iodide was added to stain the dead cells. To gate the total cell population excluding cell debris, unstained cells were acquired through flow cytometer; forward scatter (FS) and side scatter (SC) were adjusted. Cell population was gated in the FS versus SC dot plot which excluded polyploidy and cell debris. To detect the PI stained dead cells, FL2 detector was used that detects red fluorescent-labeled cells.

### Protein immunoblotting

Cellular proteins were isolated by lysing cells in extraction buffer (66 mM Tris pH ^7.4^, 2% SDS) in the presence of a protease inhibitor cocktail that contains a mixture of serine, cysteine, aspartic protease and aminopeptidase inhibitors (Cat: P8340, SIGMA-ALDRICH, USA) and incubating on ice for 15 min followed by brief sonication. The lysate was centrifuged at 10,000 g (4°C) for 30 minutes and the supernatant containing soluble proteins was collected. Proteins were quantified with the BCA™ Protein Assay Kit (Thermo Scientific, USA) using BSA as a standard.

Cell lysates were denatured in loading dye (2%SDS, 10 mM DTT, 60 mM Tris pH ^8.0^, 1% bromophenol blue and 15% glycerol) before resolving by SDS-PAGE. Following electrophoresis the separated proteins were transferred by electroblotting (40 V, 12 hours at 4°C) onto a PVDF or nitrocellulose membrane. The membrane was blocked in 1X TTS (10 mM Tris & 0.15 M NaCl, pH ^7.6^) and 5% non-fat milk for 1–2 hours at room temperature. After washing with wash buffer (1X TTS, 0.2% Tween 20 and 0.5% nonfat milk) the blot was incubated with the indicated primary antibodies for 2 hours at room temperature or 4°C overnight. Des1 antibody has been produced in our lab, MDM2 has been purchased from Santa Cruz Biotechnology (USA), and rests of the antibodies have been purchased from Cell Signaling Technology (USA). Cleaved Caspase-3 (Asp175) Antibody (Cell Signaling Technology, Cat# 9661) detects endogenous levels of activated caspase-3 (17 and 19 kDa) resulting from cleavage adjacent to Asp175 and this antibody does not recognize full length caspase-3. Cleaved PARP (Asp214) Antibody (Cell Signaling Technology, Cat# 9544) detects endogenous levels of the large fragment (89 kDa) of mouse PARP1 resulting from caspase cleavage. The antibody does not recognize full length PARP1 or other PARP isoforms. After several washes (3 times) the blot was incubated in secondary antibodies (anti-mouse/rabbit IgG) and detected by either enhanced chemiluminescence (Amersham ECL Plus™ Western Blotting Reagent, GE Healthcare Biosciences, USA) or a LI-COR ODYSSEY scanner (LI-COR. INC. Nebraska, USA).

### Extraction of RNA from Cultured cells

Cells were washed with sterile PBS before being lysed in Trizol Reagent (Invitrogen, Life Technologies, USA). The lysate was transferred to a centrifuge tube, 0.2 ml chloroform/1 ml Trizol was added, and the tube was centrifuged at 10,000 g for 15 minutes at 4°C. The supernatant was transferred to a fresh tube and the RNA was precipitated at room temperature for 10 minutes by adding 0.5 ml isopropanol/1 ml Trizol followed by centrifugation at 10,000 g at 4°C for 10 minutes. RNA pellets were washed once with 75% ethanol, resuspended in DEPC treated dH_2_O, and treated with Rnase free Dnase. RNA was then purified using the QIAGEN RNAeasy column and stored at −80°C.

### Real Time PCR

Total RNA was reverse transcribed to 1^st^ strand cDNA which was used as a template for quantitative PCR to determine the expression of several genes in MEFs. 1^st^ strand cDNA was synthesized using the iScript™ Select cDNA Synthesis Kit (Cat: 170-8897. BIO-RAD, USA). 2 µg of RNA was mixed with 2 µl of oligo(dT)_20_ primer mix (purified oligo(dT)_20_ primer in a proprietary enhancer solution) in 20 µl reaction volume along with 1x iScript select reaction mix and 1 µl of iScript reverse transcriptase. The reaction mixture was incubated at 37°C for 1 hour and then 75°C for 10 minutes to inactivate the reverse transcriptase. This cDNA was used as a template for real time PCR.

Real time PCR was performed using SsoFast™ Eva Green® Supermix Kit (Cat: 172-5200. BIO-RAD) according to the manufacturer's instructions. Briefly, 1 µl of 1^st^ strand cDNA (from previous reaction), 500 nM of each set of primers, and 10 µl of SsoFast™ Eva Green® were mixed and topped up to 20 µl with nuclease-free water. Amplification and quantification was done using BioRad CFX or Rotor-GeneQ (QIAGEN, Germany) thermal cycler. Primer sequences for the different genes are stated in Table 1.

**Table 1:** List of primers used.

Gene	Primers
β-actin	5′-TGGCATTGTTACCAACTGGG-3′
β-actin	5′-GGGTCATCTTTTCACGGTTG-3′
SPT1-For	5′-TACTCAGAGACCTCCAGCTG-3′
SPT1-Rev	5′-CACCAGGGATATGCTGTCATC-3′
SPT2-For	5′-GGAGATGCTGAAGCGGAAC-3′
SPT2-Rev	5′-GTATGAGCTGCTGACAGGCA-3′
CerS1-For	5′-CTGTTCTACTTGGCCTGTTG-3′
CerS1-Rev	5′-TCATGCAGGAAGAACACGAG-3′
CerS2-For	5′-TCTTCTCAAAAAGTTCCGAG-3′
CerS2-Rev	5′-AGTGATGATGAAAACGAATGG-3′
CerS3-For	5′-TGGCTGCTATTAGTCTGATG-3′
CerS3-Rev	5′-TCAGGATAAAGTAACCCCAG-3′
CerS4-For	5′-TGTCGTTCAGCTTGAGTGAG-3′
CerS4-Rev	5′-AGCAGGCTTCACAGAATTTC-3′
CerS5-For	5′-CTCCAACGCTCACGAAATTC-3′
CerS5-Rev	5′-ATGCAGACAGAAGATGAGTG-3′
CerS6-For	5′-GTTCGGAGCATTCAACGCTG-3′
CerS6-Rev	5′-CTGAGTCGTGAAGACAGAGG-3′
Des1-For	5′-CACCGGTACCTCGGAGCGGA-3′
Des1-Rev	5′-GTTTGGGATTGATGAACAGGGGT-3′

### Lipidomic profiling

To quantify levels of specific lipids, cells were harvested, washed with ice-cold PBS, resuspended in 900 µl of ice-cold chloroform: methanol (1∶2), and incubated in ice for 15 minutes with vortexing every 5-minutes. Four hundred microliters of ice-cold dH_2_O and 300 µl of ice-cold chloroform were added to the samples, which were then centrifuged at 8000 g for 2 min at 4°C. Lower organic phase was transferred into a clean microfuge tube. The collected samples were dried under a stream of nitrogen and stored at −80°C for LC/MS analysis as described previously [22].

### JC-1 staining to measure mitochondrial membrane potential

Cells were grown on coverslips for 16 to 18 hours and the medium was replaced with 10 µg/ml JC-1 (dissolved in DMSO) containing 1× Assay buffer (JC-1 Mitochondrial Membrane Potential Detection Kit, Cell Technology Inc. USA). The cells were placed back into the incubator (37°C, 7.5% CO_2_) for 10 minutes followed by washing with HBSS (2 times) to remove unbound dye. Monomeric JC-1 was detected by excitation at 488 nm, and emission at 527 nm. Aggregated JC-1 was detected by excitation at 543 nm and emission at 570 nm using confocal microscopy (Carl-Zeiss, Germany). Fluorescence intensity has been measured using LSM software ZEN 2009 (Carl Zeiss, Germany).

## Results

### Des1 ablation alters the cellular sphingolipidome

**Figure 1:**
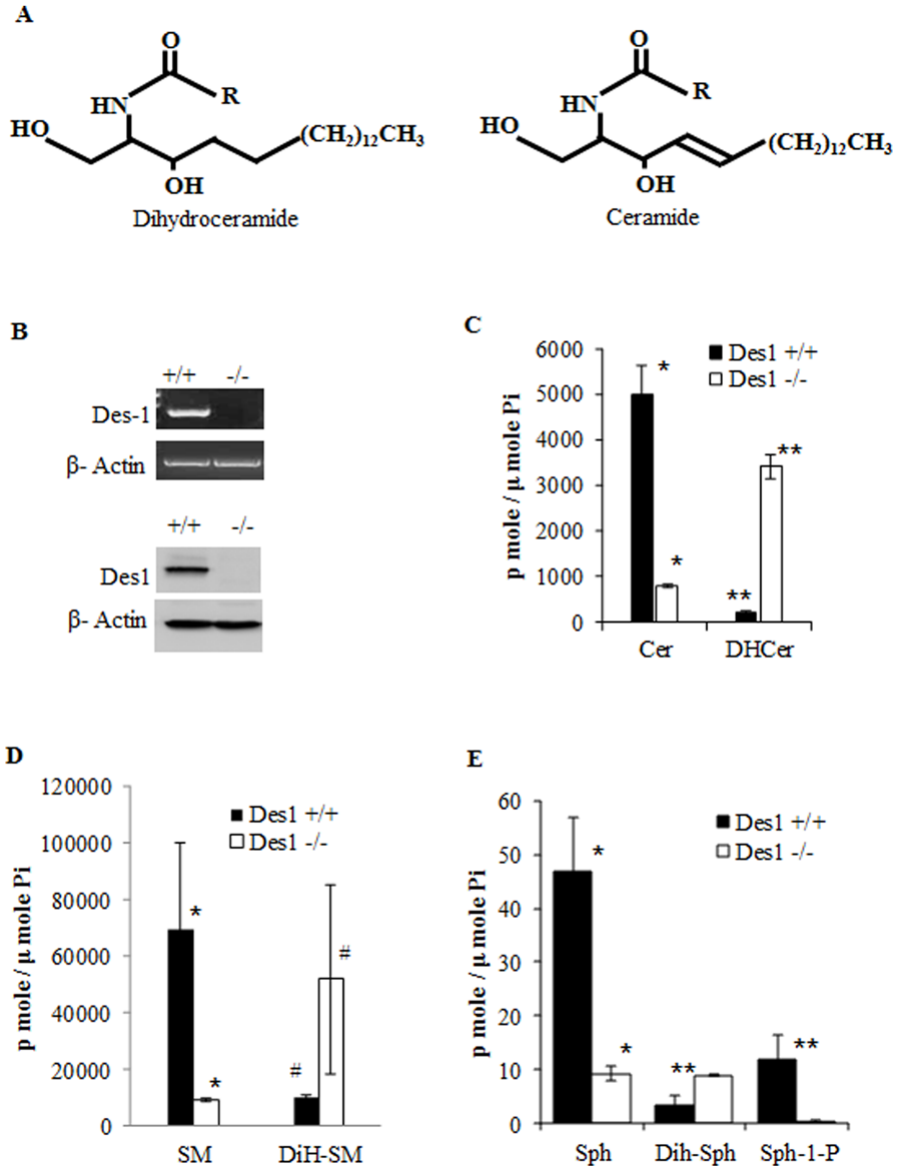
Status of ceramide and dihydroceramide to confirm the acceptability of our in vitro model. **A.** Structure of dihydroceramide and ceramide. **B.** Des 1 knock out MEFs do not express Des1 mRNA (upper panel) as well as Des1 protein (lower panel). **C.** Des1 deletion inhibits ceramide formation and causes accumulation of dihydroceramide (*P<0.01, **P<0.003). **D.** To confirm the efficiency of *Des1* ablation, downstream metabolites of ceramide and dihydroceramide were detected. Ceramide containing *Des1 +/+* cells contain sphingomyelin while the *Des1 −/−* contain mostly its dihydro-species, dihydrosphingomyelin (*P<0.05, # not significant). **E.** A similar pattern was observed for levels of sphingosine and dihydrosphingosine (*P<0.01, **P<0.05). The data represent the average values of experiments which have been done at least in triplicates. For all panels, paired t-test has been used for statistical analysis.

Des1 is the key enzyme that introduces a critical double bond in the fatty-acyl chain of dihydroceramide and converts it to ceramide in the final step of *de novo* ceramide biosynthesis (Fig. 1A). The absence of Des1 in the knockout fibroblasts was confirmed by RT-PCR and Western blotting (Fig. 1B, upper and lower panel, respectively). Moreover, microarray studies identified *Des1* as the most markedly altered gene in these two cell lines (data not shown). To assess the effect of *Des1* ablation on the sphingolipidome, lipids were quantified by LC/MS. Des1 knockout (*Des1−/−*) fibroblasts had close to 80% of the sphingolipidome replaced with dihydro-sphingolipids (i.e. sphingolipids lacking the characteristic double bond). Thus, ceramide was replaced with dihydroceramide (Fig. 1C), sphingomyelin with dihydrosphingomyelin (Fig. 1D), and sphingosine with sphinganine (Fig. 1E).

**Figure 2:**
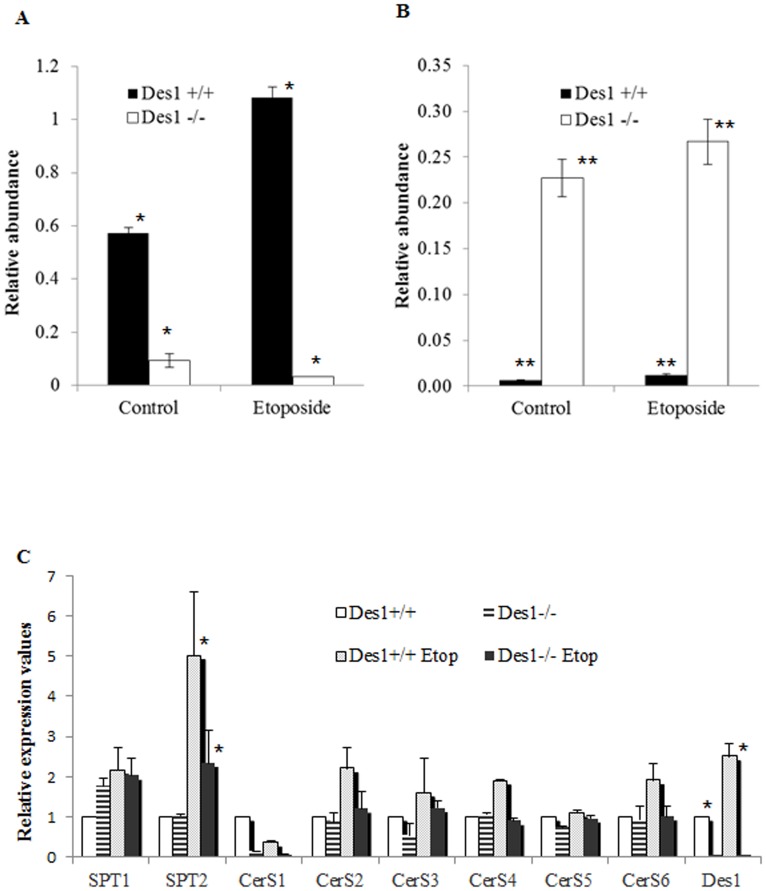
Effect of etoposide in these cell lines. **A:** Etoposide induces ceramide production in *Des1 +/+* cells (*P<0.001, n = 3) and **B:** dihydroceramide in *Des1 −/−* cells (**P<0.005, n = 3). **C:** Expression of key enzymes involved in ceramide synthesis were investigated to confirm the elevated levels of different sphingolipids in these cells using real-time polymerase chain reaction. SPT initiates sphingolipid biosynthesis and both SPT1 and SPT2 subunits are required for this process. SPT2 mRNA level in untreated *Des1+/+* and *Des1−/−* cells is equal however, SPT2 mRNA level is significantly higher in *Des1+/+* after treating them with etoposide than *Des1−/− cells* (*P<0.05, n = 3). The mRNA levels of CerS family of enzymes (CerS1-6) that contribute to the synthesis of ceramides and dihydroceramides from sphinganine were also explored. The expression level of CerS mRNA shows that in *Des1+/+* cells, CerS 2,3,4 and 6 are relatively higher after etoposide treatment compared to the *Des1 −/−* counterparts. A slight increase of CerS2 and CerS3 mRNA has been observed in etoposide treated *Des1−/−* cells than untreated *Des1−/−* cells but the differences are not statistically significant. *Des1* mRNA level was remarkably higher in *Des1 +/+* cells after treating with etoposide (*P<0.05, n = 3).

To prime the system by increasing the production of sphingolipids, we treated a subset of cells with etoposide, a chemotherapeutic agent known to activate the *de novo* sphingolipid synthesis pathway [23]. As shown in Fig. 2, etoposide doubled levels of ceramide in the wild-type (*Des1+/+*) cells (Fig. 2A), but had virtually no effect on dihydroceramides (Fig. 2B). Moreover, etoposide increased transcript levels of a number of the biosynthesizing enzymes; including SPT1 and SPT2, certain CerS isoforms, and Des1 (Fig. 2C).

### Des1 ablation increases cell survival and protects from etoposide-induced apoptosis

**Figure 3:**
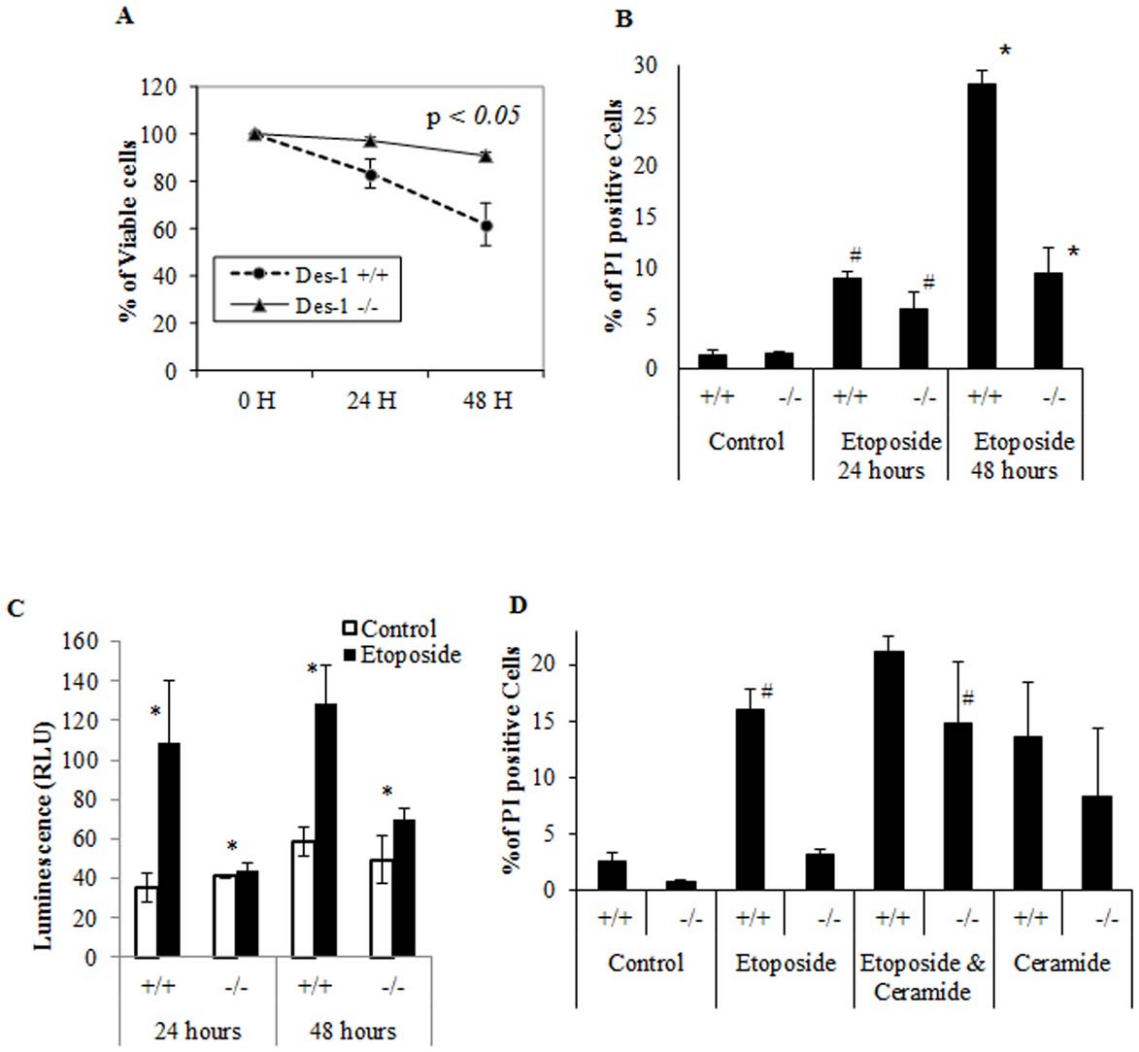
*Des1* ablation protects from etoposide-induced apoptosis. **A.** MTS assay showing percent metabolic viability after treating Des1+/+ and Des1−/− cells with 20 µM etoposide at 24 hours and 48 hours of post-treatment. MTS assay measures metabolic viability that also indicates cell viability. After 48 hours of etoposide treatment, significant increase in metabolic viability is observed in Des1−/− cells compared to Des1+/+ cells (*P<0.05, n = 3). **B.** Percentage of dead cells (PI positive cells) measured using PI (propidium iodide) staining after treating MEFs with etoposide. At 24 hours of post-etoposide treatment, percentage of PI positive cells is higher in Des1 +/+ cells compared to Des1−/− cells (# P<0.05, n = 3), However, after 48 hours significantly high cell death is observed in *Des1 +/+*cells compared to Des1−/− cells (P<0.005, n = 3). **C.** Activation of caspase 3 & 7 as measured by luminescence produced from luminogenic substrate of caspase 3&7. At both 24 hours and 48 hours, caspase 3/7 activation is significantly higher in *Des1 +/+* cells (*P<0.05, n = 3). **D.** PI staining of MEFs after treatment with 20 µM etoposide, 20 µM etoposide + 100 µM C2 ceramide, and 100 µM C2 ceramide (as a control to confirm ceramide's toxicity) for 24 hours. Addition of exogenous ceramide demise the pro-survival property of the *Des1−/−* cells in presence of etoposide (#P = 0.87, n = 3, difference is not significant) when comparing between etoposide treated Des1+/+ cells (that harbor endogenous ceramide) and Des1−/− cells treated with etoposide+exogenous ceramide (both panels are marked with #). Additional experiments with etoposide and ceramide show the efficacy of these two compounds to induce cell death (control +/+ *vs.* etoposide-treated +/+ or ceramide-treated +/+ and control−/− *vs.* etoposide-treated −/− or ceramide-treated −/−).

We next sought to understand the impact of replacing ceramides with dihydroceramides on cell survival. As shown in Fig. 3, *Des1 −/−* MEFs that contain mostly unsaturated sphingolipids were resistant to etoposide-induced cell death (Fig. 3A). We also assessed cell death by measuring staining with propidium iodide. Treatment of cells for 48 hours with etoposide killed nearly 30% of the wild-type cells, but less than 10% of the *Des1* homozygous null MEFs (Fig. 3B). Thus, Des1 ablation had a strong protective effect on cell survival. To assess whether this was due to protection from apoptosis, we measured caspase 3 and 7 activity by luminescence. Etoposide strongly activated caspase 3 and 7, and this was blunted in the knockout cells (Fig. 3C). To test whether the absence of ceramide or the presence of dihydroceramide induced apoptosis, we added back synthetic C2-ceramide (100 µM) to both the wild-type and knockout cells. Ceramide was sufficient to induce cell death, as assessed by propidium iodide staining (Fig. 3D and Fig. S1). As etoposide also induces apoptosis by inhibiting topoisomerase II, we have measures replicating cells using BrdU-FITC incorporation and no difference was observed in terms of percentage of S-phase cells (Fig. S2).

**Figure 4:**
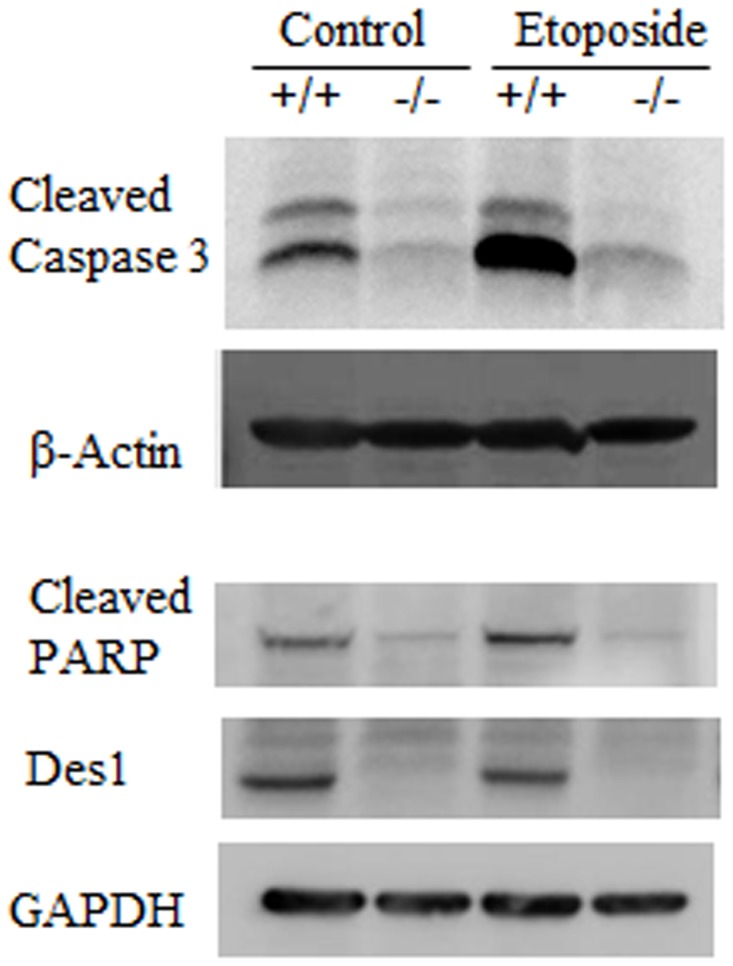
Pro-apoptotic pathway is downregulated in *Des1 −/−* cells. Cleaved caspase-3 and PARP cleavage are the hallmarks of apoptosis. After 24 hours of etoposide treatment, activation of caspase-3 and PARP cleavage was similar to untreated *Des1 −/−* cells, whereas these were markedly higher in *Des1 +/+* cells. Both antibodies detect only the cleaved forms of the proteins.

The cell survival and caspase 3/7 activity assays suggested that the knockout cells were protected from etoposide-induced apoptosis. To further validate this effect, we tested caspase 3 and PARP cleavage by Western blotting. We observed large amounts of cleaved caspase-3 in wild-type cells, and this was markedly upregulated by etoposide. By contrast, cleaved Caspase 3 was virtually undetectable in the knockout cells (Fig. 4). Similar findings were observed for PARP (Fig. 4).

**Figure 5:**
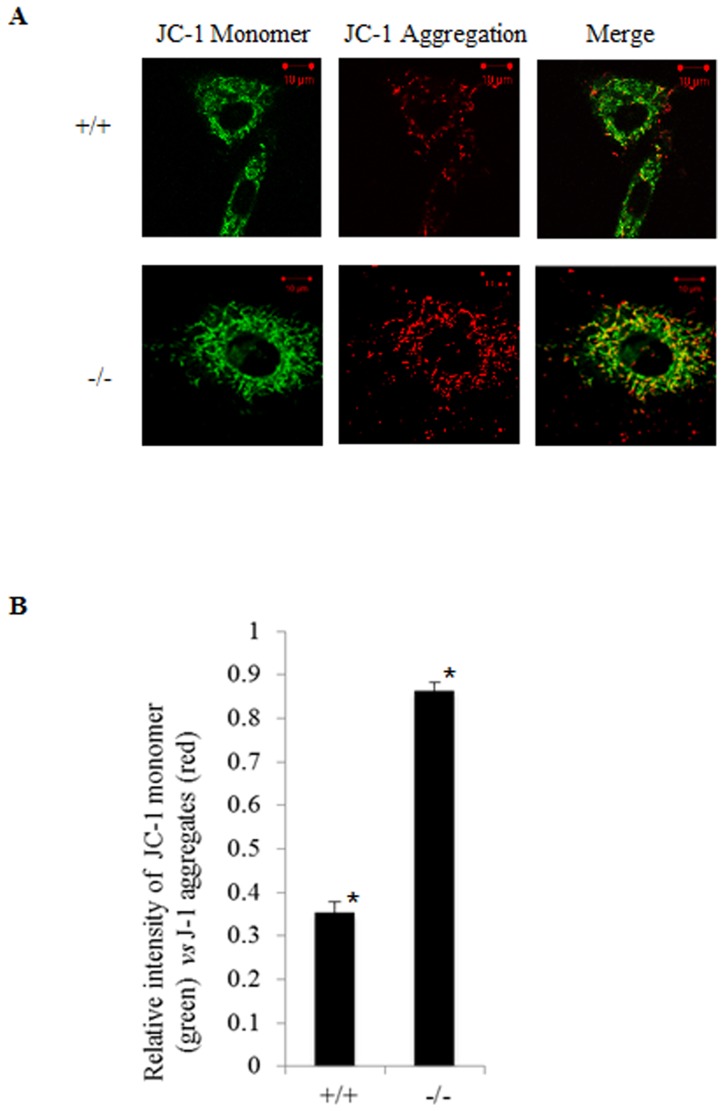
*Des1* ablation alters mitochondrial membrane potential. **A.** JC-1 staining of *Des1+/+* and *Des1−/−* indicates higher mitochondrial membrane potential in *Des1−/−* cells. Green fluorescence represents monomeric JC-1 in the cytoplasm (left panel) while red fluorescence represents J-1 aggregates accumulated in mitochondria due to high mitochondrial membrane potential (middle panel).

We additionally assessed mitochondrial membrane integrity using JC-1 staining, which has been used as an indicator of mitochondrial membrane potential in a variety of cell types. JC-1 accumulates into the mitochondria based on mitochondrial membrane potential. In the event of higher mitochondrial membrane potential, accumulation of this dye forms J-aggregates and produces red fluorescence. Depolarization of mitochondrial membrane is detected by accumulation of JC-1 monomers that produce green fluorescence. In Des1 knockout cells, we observed more J-aggregates than that in the wild type cells indicating higher mitochondrial membrane potential in these cells (Fig. 5).

### Des1 ablation activates the pro-survival kinase Akt

**Figure 6:**
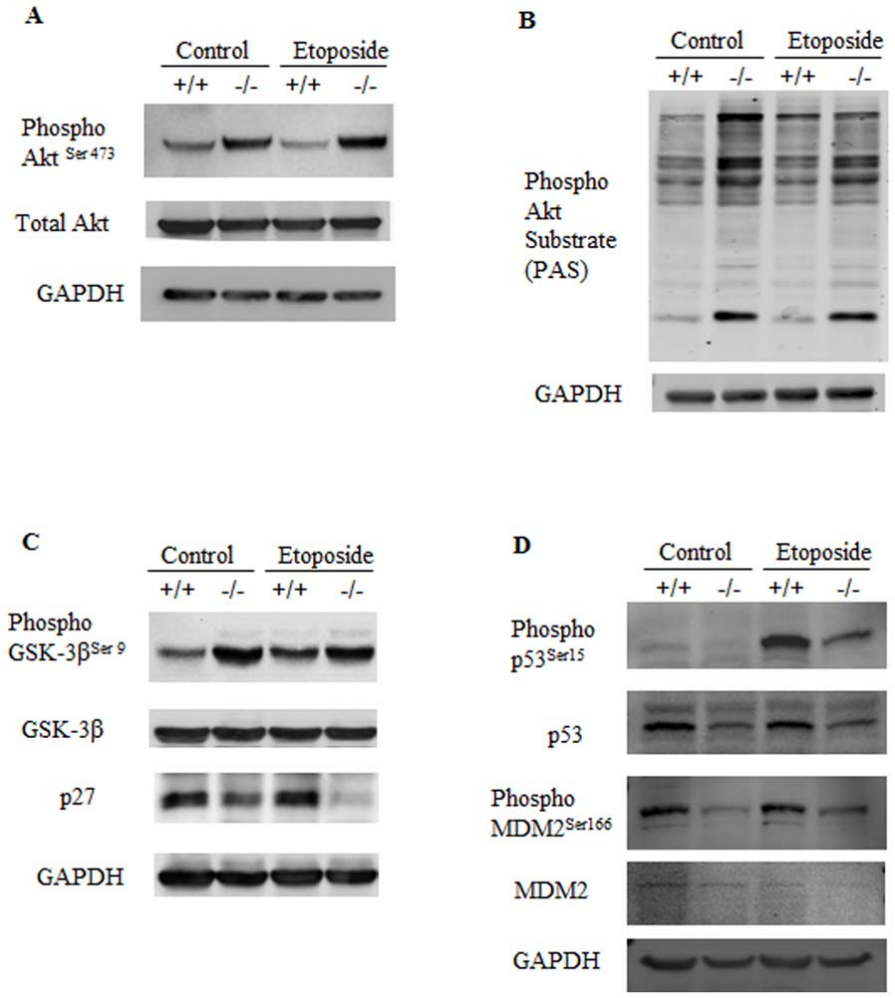
*Des1* ablation results in the activation of pro-survival kinase Akt/PKB. **A.** Phospho-Akt^Ser473^ level is upregulated in *Des1−/−* cells. **B.** Activated Akt phosphorylates its substrates at Ser/Thr. These phospho-Ser/Thr proteins detected by Phospho Akt Substrate (PAS) antibody shows increased phosphorylation in *Des1 −/−* cells. **C.** Among the Akt substrates, phospho-GSK-3β^Ser9^, the inactive form of GSK-3β, is elevated in *Des1 −/−* cells. p27Kip which is inhibited by Akt, is downregulated in *Des1 −/−* cells. **D.** Pro-apoptotic tumor suppressor p53 is phosphorylated at Ser15 residue upon genotoxic stress such as etoposide. In *Des1 −/−* cells, phospho-p53^Ser15^ and total p53 levels are significantly lower after treating with etoposide. p53 upregulates its negative regulator MDM2 at the transcriptional level. MDM2 can be phosphorylated by Akt or ERK at Ser 166 residue which in turn degrades non-phosphorylated p53. Paradoxical to the status of phospho-Akt^Ser473^, phospho-MDM2^Ser166^ is consistently higher in *Des1 +/+* cells.

Studies with ceramide analogs indicate that ceramide inhibits activation of Akt/PKB, a serine/threonine kinase implicated in cell survival. To test whether ceramide depletion led to a compensatory upregulation of Akt/PKB, we assessed its activation status by Western blotting with anti-phospho-Akt^Ser473^ antibodies. Des1 ablation markedly increased phosphorylated Akt/PKB activity in both vehicle and etoposide-treated cells (Fig. 6A). Probing with an antibody directed against the consensus Akt/PKB phosphorylation site (RXRXXS) revealed that a number of different substrates were phosphorylated in the knockout cells (Fig. 6B). Moreover, GSK-3β, a prominent Akt/PKB substrate that induces apoptosis, demonstrated increased phosphorylation on an inhibitory serine 9 residue in the knockout cells (Fig. 6C). Consistent with the observed increased Akt activation, p27kip1, another downstream target implicated in stress response and apoptosis [24], [25], was downregulated in the Des1 −/− cells (Fig. 6C).

Mdm2, another target of Akt is, normally phosphorylated at serine 166 residue and degrades unphosphorylated p53. Paradoxical to the phospho-Akt^Ser473^ status, phospho-MDM2^Ser166^ was much lower in the *Des1−/−* cells compared to the wild-type counterparts. However, elevated phospho-MDM2^Ser166^ level correlated with the total p53 level in these cells. Upon stress, such as etoposide treatment, p53 is phosphorylated at Ser15 residue which in turn activates its downstream targets to induce apoptosis. In *Des1−/−* cells, upon etoposide treatment, phospho-p53^Ser15^ level remains remarkably lower. Tumor suppressor p53 is an important mediator of apoptosis that has been implicated in etoposide-ceramide-mediated cell death [44]. Phospho-p53^Ser 15^ status in *Des1−/−* cells suggests that p53 activation could be affected in the absence of ceramides in these cells. (Fig. 6D).

### Des1 ablation downregulates PP2A

**Figure 7:**
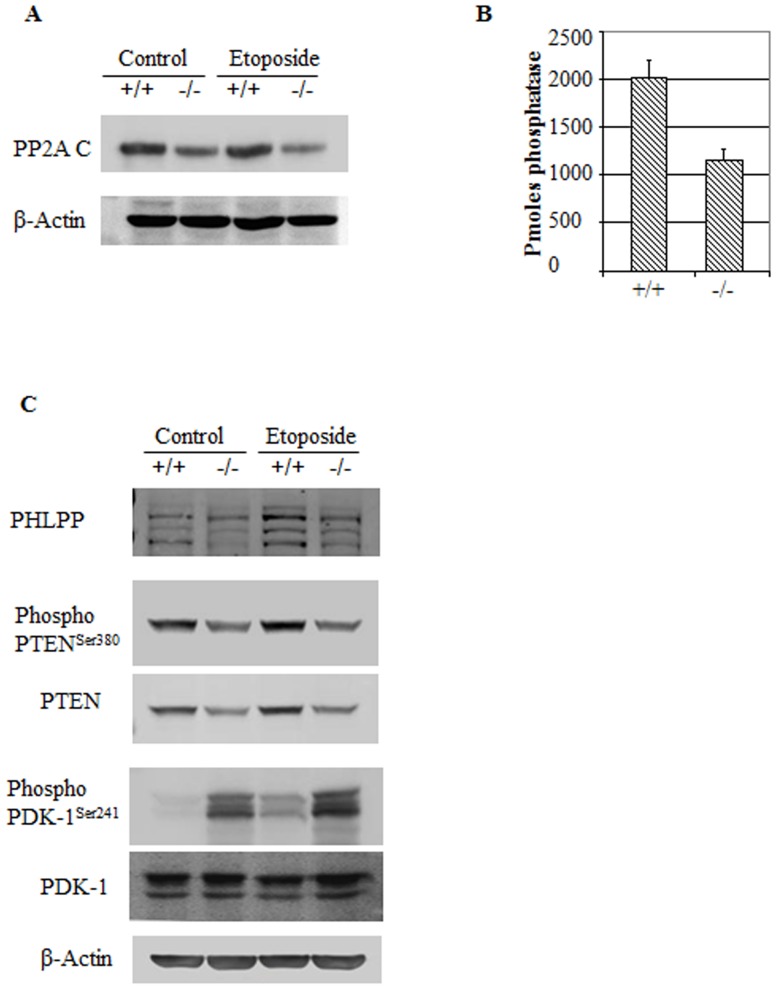
Akt/PKB negative regulators are downregulated in *Des1−/−* cells. **A.** The catalytic unit of PP2A, PP2AC, is downregulated in *Des1−/−* cells. **B.** Immunoprecipitated PP2A from *Des1−/−* cells shows lower phophatase activity compared to that of *Des1+/+* cells. **C.** Consistent with this observation, Akt inhibitors PHLPP and phospho-PTEN^Ser380^ levels are lower in *Des1 −/−* cells while the Akt activator phospho-PDK-1^Ser 241^ is elevated in these cells.

One of the prominent ways that ceramide is thought to modulate Akt/PKB is through PP2A, which dephosphorylates and inactivates the enzyme Akt [26]. Ceramide is a purported activator of PP2A [27], though intermediary proteins have also been proposed to mediate the interaction [28]. Des1 ablation decreased both the expression (Fig. 7A) and activity (Fig. 7B) of PP2A. PHLPP, a phosphatase that directly dephosphorylates Akt and promotes apoptosis [29], was downregulated in Des1−/− cells while its expression was higher in Des1+/+ cells (Fig. 7C).Surprisingly, we also saw regulation of PDK-1 that we demonstrated previously was not regulated by exogenous ceramide analogs [26]. Phosphoinositide dependent kinase-1 (PDK-1), an upstream regulator of Akt/PKB, demonstrated increased phosphorylation of an activating residue, Ser241 (Fig. 7C), while the polyphosphoinositide phosphatase PTEN, which prevents Akt/PKB activation, was downregulated to some extent (Fig. 7C).

### Des1 ablation upregulates MAPK/ERK signaling

**Figure 8:**
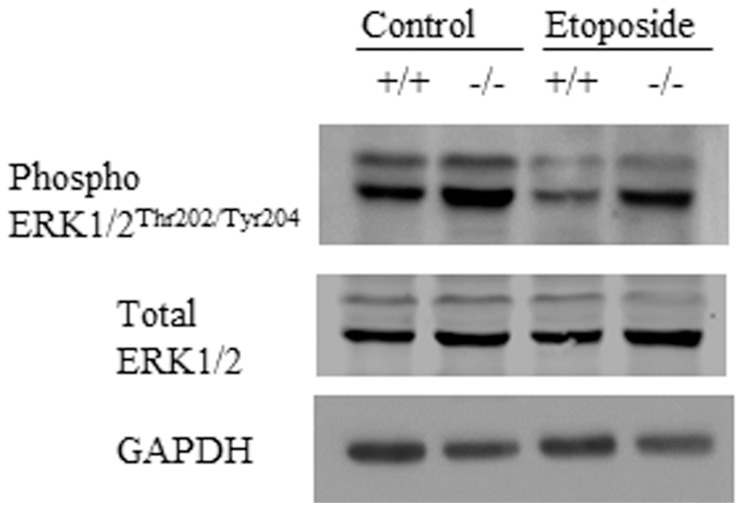
MAPK pathway is upregulated in *Des1−/−* cells. Phospho-ERK1/2 ^Thr202/Tyr204^ level remained high in *Des1−/−* cells after treating with etoposide.

Apart from PI3-kinase/Akt, the MEK/ERK pathway also plays a key pro-survival role by activating its downstream pathways that are also responsible for the regulation of a number of biological processes. Several groups have reported that PI3K plays a role in activation of the MEK/ERK pathway [30], [31]. In our study, we also investigated the status of both phosphorylated ERK1/2 (Thr202/Tyr204) and total ERK1/2 level. After etoposide treatment, we detected higher levels of phosphorylated ERK1/2 in *Des1−/−* cells whereas the total ERK1/2 level was not affected (Fig. 8).

## Discussion

Altered sphingolipid metabolism has been implicated in the etiology of several human diseases, including type II diabetes and Cushing's disease [32]. Of the myriad sphingolipids, ceramide warrants particular attention, given its increasing use as an inducer of apoptosis in cancer treatment [33], [34], [35]. Several stress signals (such as chemotherapeutic agents, ionizing radiations, inflammatory cytokines, etc.) induce long-chain ceramide accumulation in cells [36], which reduces mitochondrial mass, activates caspase-3, and induces apoptosis [37] in a number of cell types [34], [38] and through diverse pathways (e.g., Btf, p53, etc.) [39]. However, due to less-sensitive methods of detection, early reports were unable to distinguish sphingolipids and dihydrosphingolipids. Thus, the potential role for dihydroceramide, or other dihydrosphingolipids, in processes heretofore connected with ceramide is unknown.

Apoptotic agents, such as etoposide, can induce sphingolipid synthesis by increasing serine palmitoyltransferase (SPT) activation [23], [40]. Following treatment with etoposide, we observed similar phenomena, namely elevated levels of ceramide and dihydroceramide in *Des1+/+* and *Des1−/−* cells, respectively. Further, after using etoposide as an inducer of ceramide in *Des1+/+* and *Des1−/−* cells, we have observed that dihydroceramide accumulation causes upregulation of phospho-PDK1^Ser241^ and Akt/PKB, which in turn downregulates pro-apoptotic substrates. At the same time, we observed reduced expression of PP2A active catalytic subunit (PP2A C) and PHLPP, both Akt/PKB inhibitors. Concomitantly, decreased expression of active caspase 3 was detected in *Des1−/−* MEFs along with anti-apoptotic phenotypes such as resistance to etoposide and elevated phospho-Akt^Ser 473^.

Apart from inducing ceramide, etoposide also causes DNA damage and p53 activation [41], [42], [43]. The precise mechanism responsible for the down-regulation of p53 in the Des1−/− MEFs is unknown, though it likely stems from the altered ceramide/dihydroceramide ratio and subsequent effects on pro-survival pathways (e.g., upregulation of phospho-Akt^Ser 473^ and phospho-Akt substrates). In support of this view, p53 might work upstream of ceramide [44]. p53 activation induces ceramide accumulation and p53 null cells are resistant to etoposide [45]. *In vitro* expression of S1P lyase, the enzyme that degrades the pro-survival ceramide metabolite S1P, can be activated by etoposide, leading to apoptosis. This phenomenon requires p53 and p53-inducible death domain protein (PIDD) as inhibition of p53 diminishes S1P lyase-mediated apoptosis [46]. As stated earlier, we found that phospho-p53^Ser15^ level was higher in *Des1+/+* cells after treating with etoposide, which likely contributes to the increased cell death. However, in the absence of ceramide and its metabolites, dihydroceramide is not sufficient to restore this function (Fig. 6D).

Sphingolipid precursors, such as ceramide, have been associated with a wide range of human pathophysiologies, including cancer, neurodegeneration, and cardiomyopathies in which ceramides are described to be involved in modulating the DNA damage response, cell cycle progression, apoptosis and autophagy. In contrast, dihydroceramides have received less attention as in the past, these sphingolipid species were considered biologically inactive. In recent years, several reports demonstrate that this sphingolipid species are not inert but are important in mediating several cellular functions. Recent reviews by Fabrias *et al.* [47] compiled the information on dihydroceramides in response to known therapeutic agents and in the involvement of dihydrosphingolipids in several metabolic and signaling pathways. The enzymatic action of dihydroceramide desaturase can be inhibited during oxidative stress [11] and hypoxia [48] which also suggest the involvement of dihydroceramide in these cellular events. These data suggest that Des1 activity is a crucial marker for such cellular events-related disorders and Des1 could be a target to manipulate these processes. Several studies reported that the saturated fatty acid palmitate promotes insulin resistance in the heart, adipose, and skeletal muscle [49] whereas unsaturated fatty acids such as oleate have protective effects against palmitate toxicity [50]. Palmitate increases Des1 level which in turn produces ceramide and insulin resistance while oleate reduces Des1 level and prevents palmitate induced insulin resistance. This suggests that Des1 might play an important role in fatty acid-induced insulin resistance. Our recent findings propose that Des1 inhibition improves insulin sensitivity [51] and targeting this enzyme may be a possible therapeutic means for normalizing glucose homeostasis in the obese and diabetic.

This study raises an interesting possibility that altering the ceramide/dihydroceramide ratio may affect cellular susceptibility to genotoxic stress. Our data suggest that targeting *de novo* ceramide synthesis may be an effective way to prevent ceramide-mediated apoptosis. In support of this hypothesis, it has been shown that inhibition of *de novo* ceramide synthesis by myriocin or fumonisin B1 prevents cell death in the presence of excessive fatty acid [52]. Knock down of genes that are involved in ceramide metabolism, such as sphingosine kinase, cause a significant increase in ceramide and induce apoptosis [53]. Critically, this ceramide-dependent effect can be attenuated by inhibiting *de novo* ceramide synthesis [53]. Collectively, our data suggest that dihydroceramide and ceramide play opposing roles in cells, preventing or eliciting apoptosis, respectively.

## Supporting Information

Figure S1 **Histogram showing PI positive cells after treating with etoposide and/or C2 ceramide.** PI staining of MEFs after treatment with 20uµM etoposide, and 20 µM etoposide+100 µM C2 ceramide, and 100 µM C2 ceramide (as a control to confirm ceramide's toxicity) for 24 hours. The results has been summarized in figure 3D. Addition of exogenous ceramide demise the pro-survival property of the Des1−/− cells in presence of etoposide (#P = 0.87, n = 3, difference is not significant) when comparing between etoposide treated Des1+/+ cells (that harbor endogenous ceramide) and Des1−/− cells treated with etoposide+exogenous ceramide (both panels are shown in red box). Additional experiments with etoposide and ceramide show the efficacy of these two compounds to induce cell death (control +/+ *vs.* etoposide-treated +/+ or ceramide-treated +/+ and control −/− *vs.* etoposide-treated −/− or ceramide-treated −/−).
(TIF)

Figure S2 **BrdU staining to detect proliferating cells.** FITC-BrdU Flow Kits (BD Pharmingen™, USA) has been used to measure replicating cells following manufacturer's protocol. Briefly, Des1+/+ and −/− cells were plated and grow them overnight. Cultures were incubated with 10 µM BrdU for 1 hour and 2 hours. Cells were trypsinized and washed twice with cold PBS. 1.5×106 cells from each experimental unit were fixed and permeabilized in 100 µl of BD Cytofix/Cytoperm Buffer for 15 minutes at room temperature. The cells were washed once with BD Perm Wash Buffer and incubated in 100 µL BD Cytoperm Plus Buffer for 10 min on ice. The cells were then washed and fixed for 5 min at room temperature followed by Dnase treatment (300 µg/ml) for 1 hour at 37°C. This step allows exposure of BrdU that incorporates to the DNA of the replicating cells. Cells were then incubated with anti-BrdU-FITC antibody for 20 minutes at room temperature followed by staining with 7-ADD for 20 minutes at room temperature. The cells were then resuspended in 1 ml of staining buffer and analyzed for FACS. To gate, unstained cells were acquired through flow cytometer; forward scatter (FS) and side scatter (SC) were adjusted that excluded polyploidy and cell debris. To detect BrdU-FITC and 7-ADD labeled cells, FL1 and FL3 detectors were used respectively. BrdU incorporates to newly synthesized DNA in S-phase cells that are detected by anti-BrdU-FITC antibody and produces green fluorescence are shown in the box. The results in the bar diagram are expressed as percentage of the total cells in S-phase (n = 3). No significant difference is observed between Des1+/+ and Des1−/− cells in terms of the cells entering into S-phase (*P = 0.3, **P = 0.7). Representative figures are shown as do plots.
(TIF)
