# Supplementary figures and images for: Ablation of Dihydroceramide Desaturase Confers Resistance to Etoposide-Induced Apoptosis In Vitro
Source: PLoS One. 2012 Sep 11;7(9):e44042. doi: 10.1371/journal.pone.0044042 (PMC3439484; doi:10.1371/journal.pone.0044042)

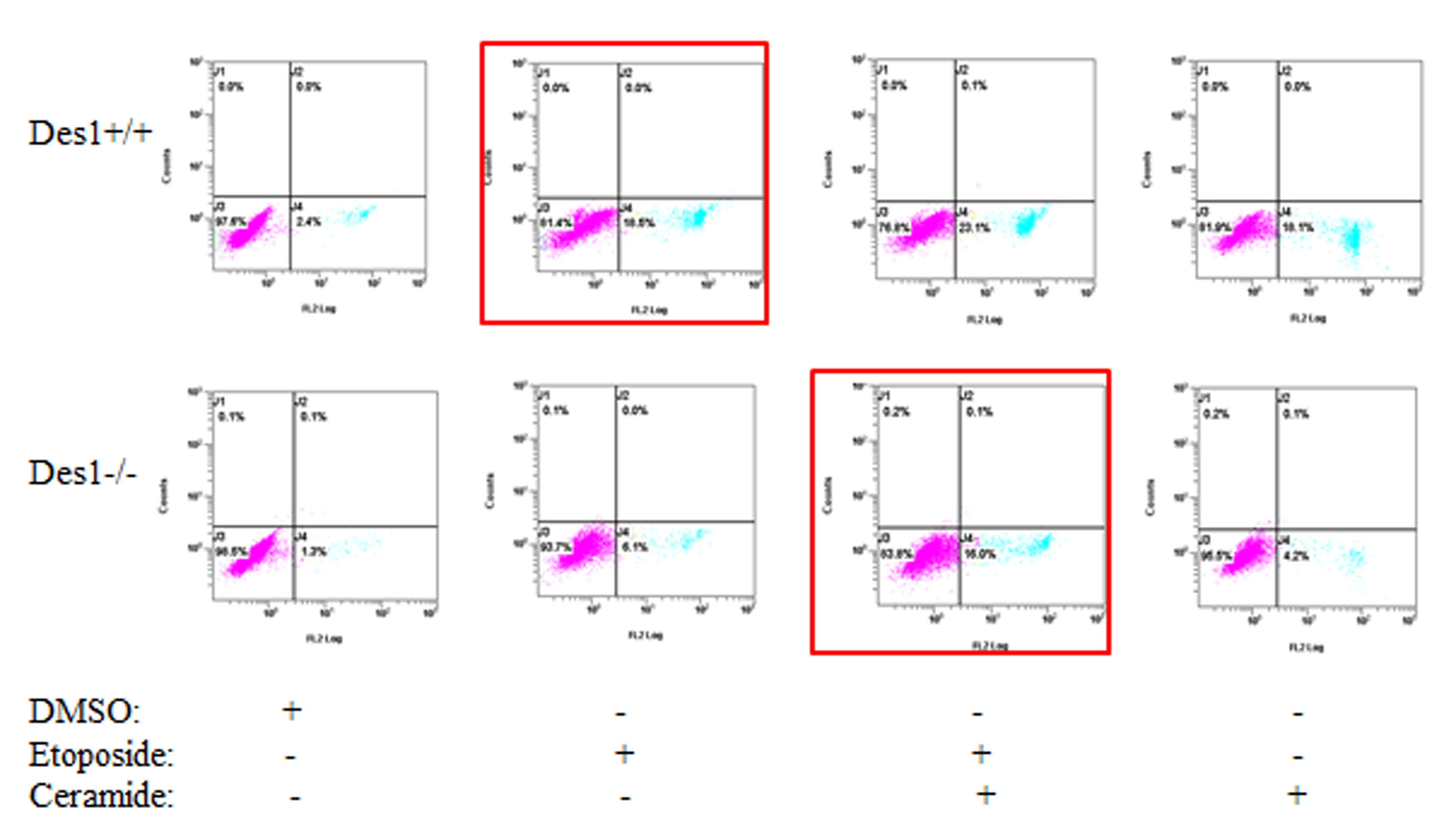

Supplement: Figure S1 — Histogram showing PI positive cells after treating with etoposide and/or C2 ceramide. PI staining of MEFs after treatment with 20uµM etoposide, and 20 µM etoposide+100 µM C2 ceramide, and 100 µM C2 ceramide (as a control to confirm ceramide's toxicity) for 24 hours. The results has been summarized in figure 3D. Addition of exogenous ceramide demise the pro-survival property of the Des1−/− cells in presence of etoposide (#P = 0.87, n = 3, difference is not significant) when comparing between etoposide treated Des1+/+ cells (that harbor endogenous ceramide) and Des1−/− cells treated with etoposide+exogenous ceramide (both panels are shown in red box). Additional experiments with etoposide and ceramide show the efficacy of these two compounds to induce cell death (control +/+ vs. etoposide-treated +/+ or ceramide-treated +/+ and control −/− vs. etoposide-treated −/− or ceramide-treated −/−). (TIF) [file pone.0044042.s001.tif]

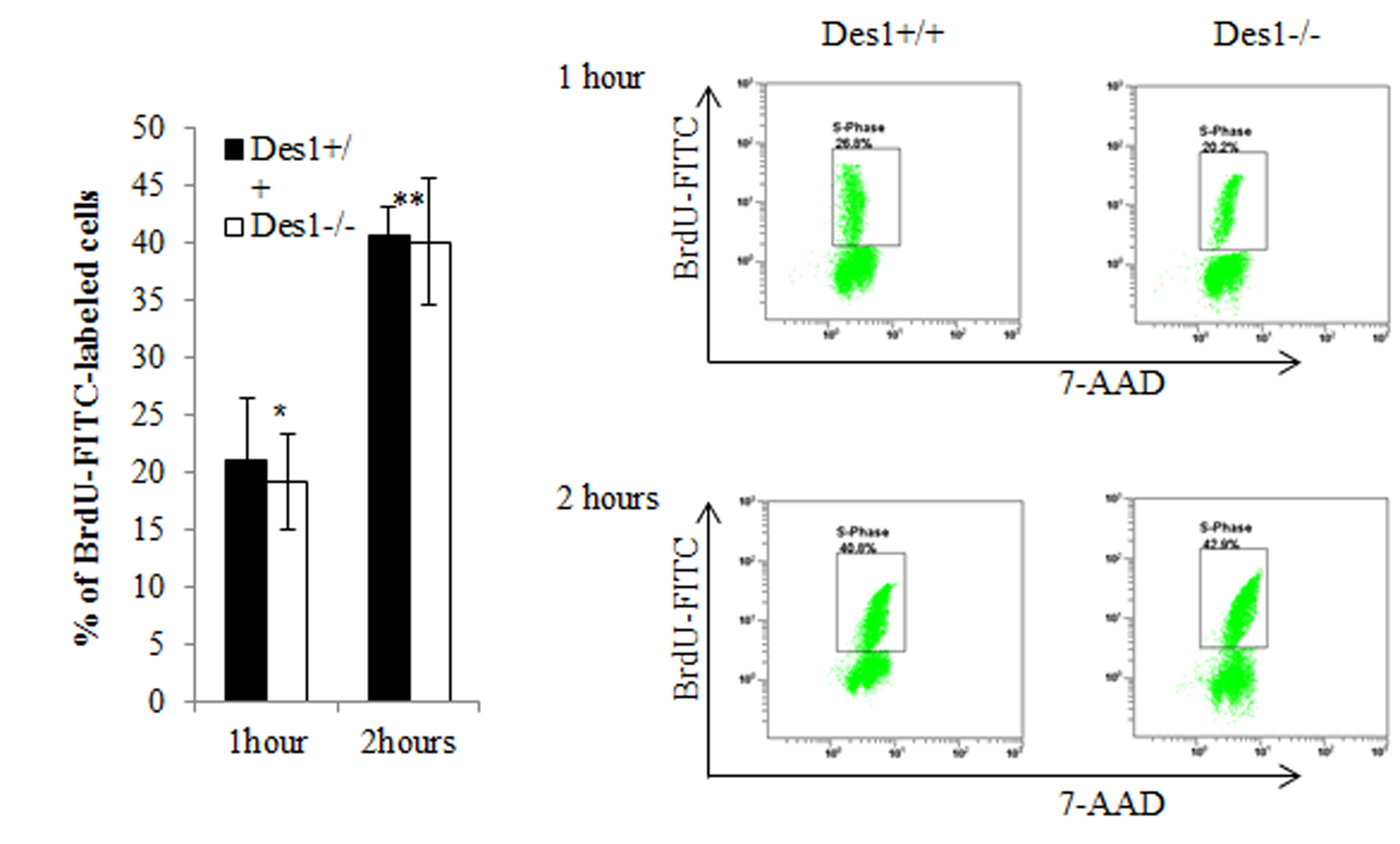

Supplement: Figure S2 — BrdU staining to detect proliferating cells. FITC-BrdU Flow Kits (BD Pharmingen™, USA) has been used to measure replicating cells following manufacturer's protocol. Briefly, Des1+/+ and −/− cells were plated and grow them overnight. Cultures were incubated with 10 µM BrdU for 1 hour and 2 hours. Cells were trypsinized and washed twice with cold PBS. 1.5×10^6^ cells from each experimental unit were fixed and permeabilized in 100 µl of BD Cytofix/Cytoperm Buffer for 15 minutes at room temperature. The cells were washed once with BD Perm Wash Buffer and incubated in 100 µL BD Cytoperm Plus Buffer for 10 min on ice. The cells were then washed and fixed for 5 min at room temperature followed by Dnase treatment (300 µg/ml) for 1 hour at 37°C. This step allows exposure of BrdU that incorporates to the DNA of the replicating cells. Cells were then incubated with anti-BrdU-FITC antibody for 20 minutes at room temperature followed by staining with 7-ADD for 20 minutes at room temperature. The cells were then resuspended in 1 ml of staining buffer and analyzed for FACS. To gate, unstained cells were acquired through flow cytometer; forward scatter (FS) and side scatter (SC) were adjusted that excluded polyploidy and cell debris. To detect BrdU-FITC and 7-ADD labeled cells, FL1 and FL3 detectors were used respectively. BrdU incorporates to newly synthesized DNA in S-phase cells that are detected by anti-BrdU-FITC antibody and produces green fluorescence are shown in the box. The results in the bar diagram are expressed as percentage of the total cells in S-phase (n = 3). No significant difference is observed between Des1+/+ and Des1−/− cells in terms of the cells entering into S-phase (*P = 0.3, **P = 0.7). Representative figures are shown as do plots. (TIF) [file pone.0044042.s002.tif]
